# Limitations of Serum Ferritin in Diagnosing Iron Deficiency in Inflammatory Conditions

**DOI:** 10.1155/2018/9394060

**Published:** 2018-03-18

**Authors:** Axel Dignass, Karima Farrag, Jürgen Stein

**Affiliations:** ^1^Department of Medicine 1, Agaplesion Markus Hospital, Goethe University, 60431 Frankfurt am Main, Germany; ^2^Interdisciplinary Crohn Colitis Center Rhein-Main, 60594 Frankfurt am Main, Germany; ^3^Department of Gastroenterology and Clinical Nutrition, DGD Clinics Sachsenhausen, 60594 Frankfurt am Main, Germany

## Abstract

Patients with inflammatory conditions such as inflammatory bowel disease (IBD), chronic heart failure (CHF), and chronic kidney disease (CKD) have high rates of iron deficiency with adverse clinical consequences. Under normal circumstances, serum ferritin levels are a sensitive marker for iron status but ferritin is an acute-phase reactant that becomes elevated in response to inflammation, complicating the diagnosis. Proinflammatory cytokines also trigger an increase in hepcidin, which restricts uptake of dietary iron and promotes sequestration of iron by ferritin within storage sites. Patients with inflammatory conditions may thus have restricted availability of iron for erythropoiesis and other cell functions due to increased hepcidin expression, despite normal or high levels of serum ferritin. The standard threshold for iron deficiency (<30 *μ*g/L) therefore does not apply and transferrin saturation (TSAT), a marker of iron availability, should also be assessed. A serum ferritin threshold of <100 *μ*g/L or TSAT < 20% can be considered diagnostic for iron deficiency in CHF, CKD, and IBD. If serum ferritin is 100–300 *μ*g/L, TSAT < 20% is required to confirm iron deficiency. Routine surveillance of serum ferritin and TSAT in these at-risk groups is advisable so that iron deficiency can be detected and managed.

## 1. Iron Deficiency in Inflammatory Diseases

Iron deficiency is a major global health problem, representing one of the leading nonfatal disease conditions worldwide [1], and is frequently seen in everyday clinical practice [2]. Timely detection and treatment are important because of the critical role played by iron in the function of all organ systems. Despite its prevalence, however, iron deficiency is often overlooked, especially in patients with inflammatory conditions, partly due to the heterogeneity of definitions provided in clinical practice guidelines [3]. Iron deficiency can be defined as “a health-related condition in which iron availability is insufficient to meet the body's needs and which can be present with or without anaemia” [4].

Conventionally, the most well-recognized risk groups for iron deficiency are the poorly nourished, those with high iron demands, such as pregnant women or adolescents, and individuals with chronic blood loss, for instance, from heavy uterine or gastrointestinal bleeding [5]. In addition, growing attention is now being paid to the iron status of patients with inflammatory conditions, which predispose them to iron deficiency [4, 6]. The most frequent of these are chronic heart failure (CHF), chronic kidney disease (CKD), and inflammatory bowel disease (IBD).

Estimates of iron deficiency in these groups have varied widely between studies due to differing definitions and diverse patient selection criteria. Overall, however, approximately 50% of patients with CHF, 24–85% of patients with CKD, and 45% of patients with IBD are iron-deficient (Table 1).

The causes of iron deficiency in these conditions are multiple and vary between individuals [2, 4, 5] (Table 1). Patients with chronic illnesses may have a poor appetite and inadequate dietary iron intake. This can be exacerbated by impaired iron absorption from the intestinal lumen caused by medications such as proton pump inhibitors [7], histamine-2 receptor antagonists [7], and calcium-based phosphate binders [8], while antiplatelet therapy can increase the risk of gross gastrointestinal blood loss [9]. Blood loss from frequent blood sampling, from gastrointestinal bleeding in IBD, or during dialysis in CKD patients can also contribute. In addition to these patient-specific etiologies, CHF, CKD, and IBD share the common effect of an ongoing inflammatory stimulus. In these chronic conditions, high hepcidin levels can restrict the uptake of dietary iron and, over time, lead to iron deficiency with reduced availability of iron for essential cellular functions [10] (see “*The Effect of Inflammation on Iron Homeostasis”* below).

All too often, investigation and treatment of iron deficiency are only triggered by the onset of (iron deficiency) anemia, at which point iron deficiency has become severe enough to exhaust iron stores and restrict erythropoiesis. However, iron has multiple biochemical and physiological functions other than erythropoiesis [21] and iron deficiency exerts various adverse effects that may arise either before or after the onset of anemia. As well as being critical for erythropoiesis, iron is essential for the function of key enzymes in the mitochondrial electron transport system [22], which may explain the fatigue that can develop in nonanemic iron-deficient individuals. Iron deficiency has also been associated with poor immune function [23]. There is a clear need for systematic diagnosis and correction of iron deficiency in inflammatory conditions.

In normal circumstances, iron status can usually be assessed adequately by measuring serum levels of ferritin. In the presence of proinflammatory stimuli, however, the diagnosis of iron deficiency is more complex. Understanding the nature of serum ferritin and, particularly, how levels of serum ferritin are influenced by inflammation is key to successful diagnosis in this context.

## 2. Ferritin: The Ubiquitous Iron Storage Protein

Intracellular ferritin is a complex made up of two types of subunit, termed H (heavy chain) and L (light chain) [24]. Twenty-four subunits combine to form a shell-like molecule that incorporates a cavity that can store up to 4,500 iron atoms [25, 26]. The H chains of ferritin have ferroxidase activity and convert Fe(II) to Fe(III) as the iron is internalized within the ferritin complex [27]. Fe(III) is sequestered within the mineral core of ferritin in the form of ferric oxyhydroxide phosphate [25]. Concentration of high amounts of iron in this unreactive form within ferritin reduces the concentration of reactive intracellular Fe(II), lowering the potential for generation of oxidant species. The L-subunit promotes formation of the iron core within the ferritin shell [24]. The ratio of H-subunits and L-subunits varies between organs, with L-subunits predominating in the liver and spleen, while H-subunits predominate in the heart and kidney [28]. Production of ferritin is controlled by the iron regulatory proteins 1 and 2 (IRP1 and IRP2), which respond to a reduction in cytosolic iron by binding to 5′-iron responsive elements in ferritin mRNAs to inhibit its translation [29, 30].

Small quantities of ferritin are also present in the serum, due to secretion from macrophages, or following cell death and lysis [31]. In contrast to intracellular ferritin, serum ferritin is iron-poor and consists almost exclusively of L-subunits [32], with the addition of glycosylated subunits (G-subunits), which are similar to the L-chain [33].

Under normal conditions, levels of serum ferritin show a close correlation with iron stores in liver biopsy samples [34], the “gold standard” for measuring the amount of iron in the body. However, serum ferritin levels can be profoundly affected by the presence of inflammation, since serum ferritin is an acute-phase protein. The acute-phase response is a major physiological defense reaction, whereby the body aims to restore physiological homeostasis in the face of inflammation [35]. Serum levels of positive acute-phase proteins including ferritin, C-reactive protein (CRP), and alpha-1-acid glycoprotein (AGP) rise dramatically as part of the inflammatory response, mediated by increased expression of cytokines such as IL-6 [35–37].

Increased levels of serum ferritin as part of the acute-phase response mean that serum ferritin levels no longer correlate with iron availability in the presence of inflammation.

Assessment of patients' iron status becomes more complex under these conditions and requires wider awareness of iron homeostatic mechanisms.

## 3. Ferritin as a Component of the Iron Regulatory System in Healthy Individuals

The uptake, transport, and storage of iron are closely regulated in the body, with ferritin playing an important role. Dietary iron in the form of inorganic Fe(III) is absorbed from the intestinal lumen across the brush border of duodenal enterocytes via active uptake mechanisms that reduce Fe(III) to Fe(II). This uptake of iron from the lumen occurs via the divalent metal transporter-1 (DMT1), which is expressed on the apical membrane of the duodenal enterocytes and is closely associated with the membrane ferrireductase DCYT-B that is responsible for the reduction of Fe(III) [5]. Once within the enterocyte, Fe(II) is then exported across the basolateral membrane by the Fe(II) transporter ferroportin [29]. After export, it is reoxidized from Fe(II) to Fe(III) by the membrane-bound ferroxidase hephaestin and possibly by intestinal ceruloplasmin [38]. Fe(III) is then released into the circulation, where it binds to the iron transport glycoprotein transferrin. Transferrin has two high-affinity binding sites for Fe(III) which maintain the iron in a redox-inert state [39]. Iron is delivered by transferrin to cells by binding to transferrin receptor 1, which is expressed on the cell surface as a response to low intracellular iron levels. Circulating iron-laden transferrin binds to transferrin receptor 1, triggering endocytosis and uptake of the iron cargo. Once internalized, the iron may be transported to mitochondria for the synthesis of heme or of iron-sulfur clusters, which are essential cofactors of various enzymes or are used to synthesize other iron-containing enzymes that are important for fundamental cellular functions such as DNA synthesis or repair [39].

If not required immediately, the iron is instead safely stored within the cell in the form of ferritin [27]. The main intracellular storage compartment, where most ferritin is located, is the cytosol. In response to the cell's need, ferritin is targeted towards lysosomes for degradation by a specific cargo molecule (NCOA4) via a process called ferritinophagy [40]. The iron is then in the so-called labile iron pool, a form of readily available cytosolic iron, and can be used for cellular functions.

**Figure 1:**
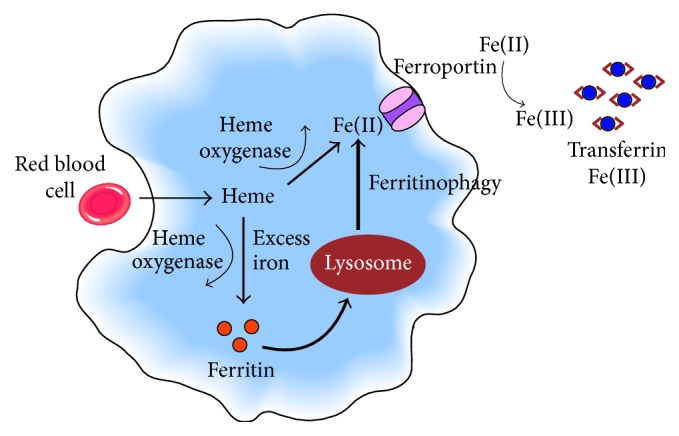
Normal iron homeostasis in the reticuloendothelial macrophage. Macrophages phagocytose aged or damaged red blood cells, using heme oxygenase 1 to release iron from heme, a recycling process that accounts for approximately 90% of the body's daily iron needs. Iron is rapidly released to circulating transferrin or, when present in excess, stored in ferritin. When required, ferritin is degraded in the lysosomes via a process called ferritinophagy and the iron is released. Iron(II) is exported from the macrophage via ferroportin in the cell membrane in a process coupled to reoxidation from iron(II) to iron(III) by membrane-bound ceruloplasmin. Iron(III) is then loaded onto transferrin for transport in the plasma.

The body's stores of ferritin are predominantly found in hepatocytes and in the macrophages of the reticuloendothelial system. Macrophages phagocytose aged or damaged erythrocytes, recycling the iron contained in heme using heme oxygenase 1 to release the iron [29] (Figure 1). This recycling accounts for approximately 90% of the body's daily iron needs, with only ~10% being met by intestinal absorption [29]. Iron is released from these storage sites as Fe(II) via ferroportin in the cell membrane. The export process is coupled to reoxidation of Fe(II) to Fe(III) by the ferroxidase enzyme ceruloplasmin and is followed by loading of Fe(III) onto transferrin for systemic distribution to other sites [26]. Transferrin saturation (TSAT) is a marker for the amount of iron available for erythropoiesis or other cellular requirements.

**Figure 2:**
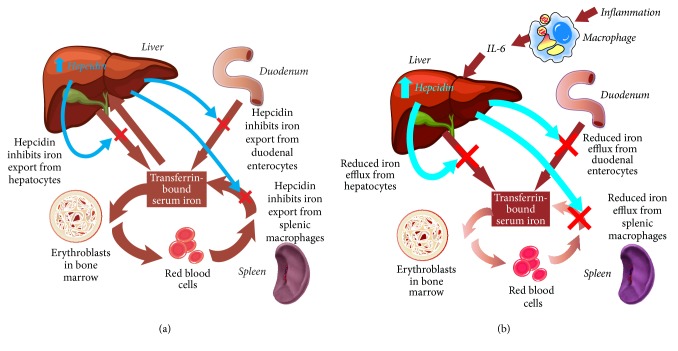
The role of hepcidin in systemic iron homeostasis. (a) In healthy individuals, hepcidin production increases in response to increasing levels of transferrin-bound serum iron and iron stores. Hepcidin internalizes and degrades the iron transporter ferroportin, restricting the export of iron from enterocytes and from iron stores in hepatocytes and macrophages, to restore normal iron levels. (b) In inflammatory conditions, hepcidin production increases in response to inflammatory cytokines such as IL-6, disrupting the usual homeostatic mechanisms. Ferroportin is internalized and degraded, reducing transmembrane export of iron, and the availability of iron to bind to transferrin is restricted.

Systemic iron homeostasis is usually maintained in the face of fluctuating dietary iron intake and varying levels of demand by regulatory mechanisms coordinated by the hepatic hormone hepcidin. Hepcidin binds to and leads to internalization and degradation of the iron exporter ferroportin. This reduces the mobilization of iron into the circulation from enterocytes and from iron stores in hepatocytes and macrophages (Figure 2(a)) [41]. In healthy individuals, increasing levels of transferrin-bound iron and elevated iron stores stimulate hepcidin upregulation, which suppresses iron export and thus lowers circulating levels of iron [29, 41]. Conversely, hepcidin production is inhibited in the presence of declining levels of iron in the circulation and in tissues or in response to other stimuli such as hypoxia and intensified erythropoiesis after blood loss [29, 41]. In this situation, reduced levels of hepcidin stimulate increased iron acquisition and release by the enterocytes in the duodenum and efflux of ferritin-bound iron from storage sites to normalize iron availability and meet increased erythroid needs.

## 4. The Effect of Inflammation on Iron Homeostasis

Patients with inflammatory conditions may have diminished iron stores, a situation described as “absolute iron deficiency.” As in patients without inflammation, this can arise due to low dietary iron intake, poor iron absorption, and/or blood loss (Table 1). In some cases, however, there may be adequate iron stores, with normal levels of serum ferritin, but insufficient iron is delivered by transferrin to meet cells' demand, a situation termed “functional iron deficiency” [42].

Functional iron deficiency (or iron-restricted erythropoiesis) in inflammatory conditions is caused by elevated hepcidin levels, triggered by inflammatory cytokines such as IL-6 [41]. The consequent internalization and degradation of ferroportin lowers the amount of iron available for binding to transferrin. Accordingly, TSAT is reduced (Figure 2(b)).

The increase in hepcidin levels in the presence of inflammation can be profound. Normal serum hepcidin values are gender-specific, with one large sample reporting median levels of 11.4 ng/mL in premenopausal women, 23.7 ng/mL in postmenopausal women, and 21.8 ng/mL in men [44]. Although ELISA shows considerable variation [45], making comparisons difficult, studies in patients with inflammatory conditions have indicated far higher levels of hepcidin: mean values up to 98 ng/mL have been reported in patients with mild CHF [46], 270 ng/mL in CKD stages 2–4 [47], and 577 ng/mL in active IBD [48]. There is evidence that levels of hepcidin correlate with the inflammatory marker CRP [44, 49] but the relation between hepcidin levels and the severity of inflammatory diseases is complex, with factors such as levels of stored iron and anemia playing a role.

Other mechanisms can also affect iron homeostasis in the presence of inflammation [50]. These include downregulation of transferrin expression by hepatocytes in response to increased levels of circulating IL-6 and other proinflammatory cytokines [51] and suppression of ferroportin mRNA [52, 53].

## 5. Serum Ferritin and TSAT as Complementary Markers of Iron Status in Inflammatory Conditions

Since serum ferritin level rises as part of the acute-phase response, measurement of serum ferritin alone cannot exclude iron deficiency in patients with CHF, CKD, or IBD. Additional testing is required usually by the assessment of TSAT.

Both serum ferritin and TSAT tests are readily available and inexpensive. There are certain limitations to both types of tests, however. Serum ferritin levels in normal individuals have been reported to vary by 15% in men and 27% in women on a day-to-day basis [54], with variations of up to 62% when measured over a longer term [55]. Additionally, serum ferritin assays differ in terms of the antigens used and have technical variations, for example, in calibration procedures and in the choice of reference standards [56], leading to significant analytical variability. One large-scale comparison in the USA found that interassay differences could be as high as 54% [55]. Accordingly, where serum ferritin levels are measured in different laboratories (e.g., before and after hospital discharge), a change in serum ferritin may not necessarily reflect a change in iron status. For TSAT, there is also substantial biological variability (up to 38% [57]). Serum iron levels, used to calculate TSAT, show diurnal fluctuation [58] and are influenced by oral iron supplements and the amount of iron in the diet [59]. Serum iron should generally be measured in the morning on an empty stomach to minimize variation. As well as physiological alterations, there is a degree of interassay variability for serum iron measurements, which are the basis for calculation of the TSAT level. One study of 10 ferritin assays and five TSAT assays, which analyzed samples from 114 patients on hemodialysis, found 63% variation in the reported levels of serum ferritin but only 10% variation for TSAT [56].

One practical caveat to note is that laboratory reports usually include a reference range of “normal” serum ferritin levels. These ranges can vary, and the “normal” range is both assay-dependent and laboratory-dependent. However, the “normal” ranges are all based on healthy patients without elevated inflammatory cytokine levels and should not be applied to patients with inflammatory conditions.

## 6. Diagnostic Thresholds for Serum Ferritin and TSAT in Inflammatory Conditions

In the general population, WHO defines low serum ferritin as <15 *μ*g/L in adults and <12 *μ*g/L in children [43]. A level of 30 *μ*g/L has been identified as the most sensitive (92%) and specific (98%) cutoff level to indicate iron deficiency, correlating with the absence of iron stores in the bone marrow regardless of the presence or absence of anemia [60]. Expert guidelines in inflammatory conditions usually specify both serum ferritin and TSAT thresholds for the diagnosis of iron deficiency [3, 61–63], but cutoff values are not consistent [3].

A simplified diagnostic approach in patients with CHF, CKD, or IBD recommends that iron deficiency be diagnosed based on the following cutoff values: serum ferritin < 100 *μ*g/L or TSAT < 20%, and if serum ferritin is between 100 and 300 *μ*g/L, a TSAT test is required to confirm iron deficiency [4] (Table 2). Hemoglobin levels may support the diagnosis of iron deficiency but do not need to be below normal to confirm the diagnosis [3]. This diagnostic approach has been used widely in recent large-scale prevalence studies of iron deficiency [11–13].

Where doubt about a patient's iron status persists despite measurement of both serum ferritin and TSAT, other tests may be required to definitively exclude iron deficiency.

## 7. Other Diagnostic Tests for Iron Deficiency

Where inflammation is present and serum ferritin with TSAT testing is inconclusive, other tests may be necessary (Table 3).

### 7.1. Hematological Markers

The percentage of hypochromic erythrocytes (% HYPO) and the content of reticulocyte hemoglobin (CHr or RetHb) are the most frequently used hematological indices of iron status.

Iron-deficient erythropoiesis increases the proportion of % HYPO, generally defined based on a mean corpuscular hemoglobin concentration (MCHC) < 280 g/L [32]. An increased level of % HYPO is regarded as a sensitive and early indicator of iron deficiency [32]. A cutoff of 6% for % HYPO has been proposed in guidelines for the management of CKD as being diagnostic for functional iron deficiency when combined with low TSAT [65, 66]. Measurements of % HYPO are sensitive to temperature, since erythroid expansion with a reduction in MCHC occurs when samples are stored at room temperature or above, so the analysis should be performed within four hours if the sample is not refrigerated [32]. This has hampered more extensive use of % HYPO as a diagnostic test [67].

CHr content provides a “real-time” indication of the functional state of bone marrow, with a value < 28 pg proposed as the cutoff point for diagnosis of iron-deficient erythropoiesis [68]. One study of 36 patients on chronic hemodialysis showed CHr to have 100% sensitivity and 73% specificity for iron-deficient erythropoiesis [69] and other trials have confirmed the predictive value of CHr in this setting [70, 71]. Decreased CHr is a convenient marker that is available via standard cell count measurements without additional costs. Preservation of samples during storage and delivery to the laboratory is again an issue, however, creating logistic challenges [67].

### 7.2. Soluble Transferrin Receptor (sTfR) and the sTfR-Ferritin Index

Soluble transferrin receptor (sTfR) is a truncated form of transferrin receptor 1 (TfR). When TfR is not stabilized by iron-laden transferrin, it is cleaved by a membrane protease in erythroid cells, releasing sTfR. Levels of sTfR increase in the presence of iron deficiency and are reduced in patients with iron overload [5, 32]. The ratio of sTfR (levels of which are high when iron stores are low but normal or low in the presence of inflammation) to log ferritin (levels of which are low when iron stores are low but normal or high in inflammatory condition) has been suggested to be a useful test and is known as the “sTfR/log ferritin index” [5, 72].

sTfR is a marker for the activity/size of the erythrocyte precursors in the bone marrow and is not directly influenced by inflammation [73]. However, production of inflammatory cytokines can inhibit erythropoiesis both directly and indirectly by suppressing erythropoietin synthesis and erythropoiesis [74], an effect that reduces levels of sTfR. sTfR concentrations can therefore remain normal despite depleted iron stores [75] and become less reliable as the degree of inflammation increases. Levels of sTfR are also affected by age and ethnicity and by altitude, complicating interpretation [74, 76]. Since sTfR is a marker of erythropoietic activity, levels increase after administration of ESAs [77]. Various commercial immunoassays have been developed for the assessment of sTfR, but practical issues such as variations in assay types and lack of standardization mean that neither sTfR nor the sTfR-ferritin index are widely used [5, 32, 74]. They tend to be applied when automated red cell parameters such as CHr or % HYPO are not available [68].

### 7.3. C-Reactive Protein

Assessing the severity of inflammation based on the level of high sensitivity CRP (hsCRP) could theoretically be helpful in order to understand the extent to which serum ferritin levels have risen as part of the acute-phase response. Recently, the effect of adjusting ferritin-based diagnostic criteria for iron deficiency according to levels of CRP or AGP was examined using data from the international Biomarkers Reflecting Inflammation and Nutritional Determinants of Anemia (BRINDA) project [78]. Researchers found that the observed rate of iron deficiency showed a clear inverse relation to CRP levels. In women of reproductive age, the incidence of iron deficiency (based on a serum ferritin threshold of 15 *μ*g/L) was 6.1% and 29.0%, respectively, in the subgroups with the highest and lowest deciles of CRP. Similar inverse associations were seen for AGP levels and in preschool children [78]. The authors proposed that corrections should be applied for CRP or AGP levels during iron deficiency surveillance programs [78].

However, there is currently no consensus on when to include CRP in the diagnostic work-up for iron deficiency or what thresholds should be applied. CRP is not included in guidelines for the evaluation of iron status in inflammatory conditions [3]. In IBD, however, measurement of CRP (with a threshold of 5 mg/L), or use of stool markers such as calprotectin or lactoferrin, has been recommended to confirm whether the disease is active or in remission, with transabdominal ultrasound or endoscopy if required [4]. This allows serum ferritin results to be interpreted accordingly, since levels are raised in active IBD [63].

## 8. Assessing High Serum Ferritin in Inflammatory Conditions

The body has no active excretion mechanism for iron and is thus vulnerable to a positive iron balance and, eventually, risk of iron overload if the homeostatic systems become disrupted or are bypassed. Excess body iron can be toxic, saturating the iron-binding capacity of transferrin and resulting in non-transferrin-bound iron [79], which can be taken up in an uncontrolled manner with the risk of organ damage in the endocrine system, heart, and liver [79].

WHO guidelines state that, in the absence of inflammation, serum ferritin > 200 *μ*g/L in men or >150 *μ*g/L in women confers a risk of iron overload in the general population [43]. In inflammatory conditions, where serum ferritin levels are raised as part of the acute-phase response, however, these thresholds do not apply and TSAT should be measured to avoid a misdiagnosis of iron overload.

Striking increases in serum ferritin levels can also occur in the event of acute inflammatory or infectious events. One case control study of 47 patients found the mean serum ferritin level to be 1000 *μ*g/L in patients with acute kidney failure compared to 90 *μ*g/L in patients with CKD [80]. The same study reported very high serum ferritin levels (mean 505 *μ*g/L) in a control group of 10 patients with normal renal function who had an acute infection. High serum ferritin levels in patients with a chronic inflammatory condition who experience an acute episode or who develop an infection should thus be interpreted particularly cautiously.

Other causes of iron overload include multiple blood transfusions, for example, in long-term treatment of transfusion-dependent anemias such as thalassemia and myelodysplastic syndromes, where hepcidin-regulated homeostatic mechanisms are bypassed. Certain genetic conditions can also lead to iron overload. The most frequent of these is hereditary hemochromatosis, in which control of iron uptake from the gut is defective due to abnormally low levels of hepcidin [81].

## 9. Special Situations Affecting Serum Ferritin Levels

Certain demographic and physical characteristics alter iron homeostasis and affect serum ferritin levels. Some of these (particularly obesity and old age) are frequent in patients with inflammatory conditions such as CHF. Obese patients are known to have an increased risk for iron deficiency [82]. Patients with high body mass index have increased levels of hepcidin [83], likely due to adiposity-related inflammation [84], resulting in restricted dietary iron absorption [85] and reduced TSAT levels [82]. As in other inflammatory conditions, serum ferritin levels are higher than in nonobese individuals [86]. Older patients are also prone to absolute iron deficiency due to factors such as an iron-poor diet and medications that inhibit dietary iron absorption [87]. Low-grade inflammation [88] is often present in older people [88–90], with the potential for functional iron deficiency. Inadequate iron for erythropoiesis, as determined by bone marrow aspirates, is frequently found even in elderly patients with serum ferritin levels up to 75 *μ*g/L [91].

Concomitant diseases can also complicate the interpretation of serum ferritin concentrations. Ferritin levels are elevated in the serum of many patients with cancer, particularly in the presence of more aggressive disease [92], due to chronic inflammatory effects as indicated by upregulation of IL-6, CRP, and hepcidin [93–95]. Patients with liver disease exhibit complex iron homeostasis disturbances [96] that become more pronounced with greater severity of disease [97]. Reduced expression of ferroportin and the consequent inhibition of iron export from hepatocytes [98] can lead to iron deposits in the liver [99], stimulating increased hepcidin production [97, 100, 101]. Hepatitis promotes an increase in serum ferritin in response to the inflammatory stimulus [99], such that functional iron deficiency can develop. In nonalcoholic liver disease, for example, approximately one-third of patients have elevated serum ferritin levels [99, 102]. Careful interpretation of serum ferritin levels in the obese and elderly and in patients with liver disease is required, and TSAT measurement should be performed before ruling out a diagnosis of iron deficiency.

## 10. Conclusions

Iron deficiency often remains undiagnosed and untreated in the context of inflammatory conditions [103]. It may not be suspected because the typical symptoms, such as fatigue, can be similar to those of the underlying disease. Even in the absence of anemia, however, iron deficiency can negatively affect patients' quality of life, and expert guidelines in CHF, CKD, and IBD recognize that iron deficiency should be detected and managed [61–63, 104]. Routine laboratory testing is advisable, with reassessment every 3 to 12 months or in the event of disease progression [4]. Measurement of both serum ferritin and TSAT offers a straightforward means to identify the presence of iron deficiency in these at-risk groups [4]. A diagnosis of iron deficiency can be made in these conditions, regardless of whether anemia is present, if serum ferritin is <100 *μ*g/L or TSAT is <20%, using TSAT to confirm iron deficiency if serum ferritin is between 100 and 300 *μ*g/L [4, 5]. This approach improves diagnostic sensitivity and allows prompt initiation of treatment. Iron replenishment can be achieved despite the presence of inflammation by use of intravenous iron therapies, as per expert guidelines [61–63, 104, 105]. The intravenous route bypasses the hepcidin-induced blockade of oral iron uptake and release and avoids the problem of intolerance to oral iron [6, 106]. Clinical trials have shown intravenous iron to achieve iron repletion more rapidly and efficiently than oral iron, including studies in patients with inflammatory conditions [107–111]. Intravenous iron should be avoided in case of potential infections.

With effective therapy available, surveillance of serum ferritin and TSAT levels in these at-risk groups is prudent so that iron deficiency can be treated before progression to symptomatic anemia or other complications. At the same time, iron overload should be avoided, and markers to be followed need to be established.

## Figures and Tables

**Table 1:** Causes, prevalence, and clinical consequences of iron deficiency in inflammatory conditions.

Disease	Key causes of iron deficiency [2, 4, 5]	Estimated prevalence	Potential clinical consequences [4]
Chronic heart failure	Inflammatory state Loss of appetite/poor nutrition Reduced iron uptake from GI tract due to edema or common concomitant medications (e.g., histamine-2 receptor antagonists, calcium-based phosphate binders, antiplatelet therapies, and proton pump inhibitors)	~50% (range: 37–63%) [11–14]^a^	Fatigue and reduced exercise capacity, work capacity, and quality of life Associated with increased hospitalization and mortality

Chronic kidney disease	Inflammatory state Reduced hepcidin excretion by the kidneys Blood loss from dialysis sessions Chronic intestinal bleeding (e.g., platelet dysfunction) Frequent phlebotomy Acute expansion of erythroid mass under ESA therapy Poor appetite	24–85% (highest incidence with more severe CKD) [15–17]^b^	Iron-deficiency anemia associated with fatigue, increased mortality, and progression to end-stage renal disease

Inflammatory bowel disease	Inflammatory state Chronic blood loss from the GI tract Poor appetite Reduced uptake of iron from the GI tract Bowel resection	~45% (range: 43–55%) [18–20]^c^	Fatigue, exhaustion, reduced exercise capacity and quality of life

CKD, chronic kidney disease; ESA, erythropoietin-stimulating agent; GI, gastrointestinal. ^a^Iron deficiency defined as serum ferritin < 100 *μ*g/L or 100–300 *μ*g/L [11–13] (or <800 *μ*g/L [14]) with transferrin saturation (TSAT) < 20%. ^b^Iron deficiency defined as serum ferritin < 100 *μ*g/L or TSAT < 20%. ^c^Iron deficiency defined as serum ferritin < 30 *μ*g/L or TSAT < 16% [18, 19] or <20% [20] or as serum ferritin < 100 *μ*g/L if C-reactive protein (CRP) > 5 mg/L [20] or >10 mg/L [18].

**Table 2:** Proposed serum ferritin and TSAT thresholds for the diagnosis of iron deficiency in patients with or without inflammatory conditions.

Population	Thresholds
No inflammatory condition [43]	Serum ferritin < 30 *μ*g/L (N.B.: false negatives are common)

Inflammatory conditions [4, 5]	Serum ferritin < 100 *μ*g/L *or* TSAT < 20% If serum ferritin is 100–300 *μ*g/L, a TSAT test is required to confirm iron deficiency

TSAT, transferrin saturation.

**Table 3:** Laboratory tests for iron deficiency [4, 32, 50, 64].

	Absolute iron deficiency	Functional iron deficiency in inflammation	Both absolute iron deficiency and functional iron deficiency
Serum ferritin	↓	↑	Depends on the degree of iron deficiency
TSAT	↓	↓	↓
Hepcidin	↓	↑	Depends on degree of iron deficiency
CHr	↓	↓	↓
% HYPO	↑	↑	↑
sTfR	↑	↓	↓ or normal
sTfR/log ferritin	↑	↓	↑
CRP	Normal	↑	↑

CHr, content of reticulocyte hemoglobin; CRP, C-reactive protein; % HYPO, percentage of hypochromic erythrocytes; sTfR, soluble transferrin receptor; TSAT, transferrin saturation.
